# Characteristics associated with the use of specialized mental healthcare by Norwegian adults with intellectual disability: a cross-sectional study

**DOI:** 10.1186/s12913-026-14713-w

**Published:** 2026-07-01

**Authors:** Erlend Refseth Pedersen, Audny Anke, Monica Isabel Olsen, Erik Søndenaa

**Affiliations:** 1 Department of Mental Health, Norwegian University of Science and Technology, Olav Kyrres Gate 9, Trondheim, 7030 Norway; 2 St. Olavs Hospital, Centre for Research and Education in Forensic Psychiatry, Trondheim, Norway; 3 Department of Rehabilitation, University Hospital of North Norway, Tromsø, Norway; 4 Department of Clinical Medicine, Faculty of Health Sciences, UiT - The Arctic University of Norway, Tromsø, Norway; 5 Institute of Health and Society, Research Centre for Habilitation and Rehabilitation Model and Services (CHARM), Faculty of Medicine, University of Oslo, Oslo, Norway; 6 Department of Teacher Education and Pedagogy, Faculty of Humanities, Social Sciences and Teacher Education, UiT- The Arctic University of Norway, Tromsø, Norway

**Keywords:** Mental health, Intellectual disability, Challenging behavior, Health care services, Mental health services, Habilitation

## Abstract

**Background:**

Studies have reported unmet healthcare needs among people with intellectual disability. However, there appears to be a lack of research examining the use of mental health services. The purpose of the present study was to address this knowledge gap and examine characteristics associated with use of the services in Norway.

**Method:**

A cross-sectional community-based survey including 199 adult participants (55% men) with intellectual disabilities. Assessment comprised the POMONA-15 health indicators and instruments designed to assess aberrant behavior (The Aberrant Behavior Checklist-Community, ABC-C) and screen for possible mental health problems (Moss Psychiatric Assessment Schedules-Check, MPAS-Check).

**Results:**

During the last year, 49% of the participants had received specialist mental healthcare and/or specialized habilitation services. Further, 18% scored above threshold on the MPAS-Check, and the prevalence of challenging behavior in our sample was estimated to be 16% using the ABC-C. The presence of challenging behavior, indicated by the ABC-C, was found to significantly increase the odds of using specialist mental healthcare and/or specialized habilitation services.

**Conclusion:**

When developing policies and measures to improve access to mental health care services, it is important to consider factors that serve as barriers and facilitators to access. Our findings indicate that psychotropic medication is primarily managed by general practitioners and suggest a potential gap in follow-up of these medications by specialist mental health and specialized habilitation services.

## Introduction

Adults with intellectual disability have reduced access to healthcare services, and healthcare specialists in particular, compared to the general population [1–4]. In a meta-analysis by MG Mazza, A Rossetti, G Crespi and M Clerici [5] the overall reported prevalence of psychiatric disorders among adults with intellectual disability was 34%. The authors concluded that the elevated prevalence of psychiatric disorders among adults with intellectual disability underscores the importance of systematic identification and diagnosis of these conditions within this population. However, people with intellectual disability and mental health problems have experienced restricted availability of psychiatric evaluation, therapy, and support, as indicated by studies from Europe (including Norway), North America, and Australia [6, 7]. In Norway, some of these challenges emerged after the closure of centralized institutions for people with intellectual disability [8]. Institutional healthcare has been reduced across many countries since the normalization movement in the 1970s, and people with intellectual disability consequently more often reside in community settings [9, 10]. In Norway, the municipalities inherited the task of providing services to individuals with intellectual disability from county administration in 1991. By 1996, all institutions serving people with intellectual disability were closed [11, 12]. As a result, specialized habilitation services were developed in all counties of Norway to provide physical and mental health care to people with intellectual disability. These services are administered within the specialist health services and provide outpatient and inpatient care for people with intellectual disability. The specialized habilitation services work in close collaboration with primary health services where the general practitioner (GP) refers patients to habilitation [13].

All Norwegian citizens are entitled to healthcare, including specialized services, as part of the universal health coverage financed by taxes [14]. Still, Norwegian authorities have addressed the need for further improvement in the health services offered to people with intellectual disability in national reports from 2017 [15] after service violations were found in more than 80% of the municipalities inspected. A Norwegian green paper report from 2018 identified challenging behavior in combination with mental health problems as particularly challenging. In this report, managers in the municipalities described a lack of available medical expertise, and access to psychiatrists in particular, and the need for the strengthening of psychiatric and inpatient services for people with mental health problems, intellectual disabilities, and challenging behavior [16]. These reports resulted in the development of national guidelines, referred to as “Good health and care services for people with Intellectual disabilities”, that are currently being implemented in the services [17].

Limited research has been conducted on how the utilization of mental healthcare services is associated with levels of intellectual disability. However, previous studies have found disparities, including a scarcity of psychiatric services for people with more severe intellectual disability [18, 19]. People with intellectual disability have a higher prevalence of medical conditions [20] that have an impact on their overall well-being and mental health [21]. People with intellectual disabilities may express their reduced well-being through challenging behavior, as they may struggle to communicate their discomfort [22].

H Costello, N Bouras and H Davis [23] reported that psychiatrists’ comprehension and expertise concerning mental health problems in people with intellectual disability was inadequate in England. The study concluded that specific knowledge of this population’s unique requirements with regard to psychiatric assessment, diagnosis, and therapeutic approaches is essential [23].

Co-occurring mental illnesses may have serious consequences when it comes to treatment and health care service access for people with intellectual disability [5]. A scoping review by EL Whittle, KR Fisher, S Reppermund, R Lenroot and J Trollor [24] pointed out that despite the elevated risk of mental illness among people with intellectual disabilities, the utilization of mental healthcare services remains disproportionately low.

People with intellectual disability may encounter obstacles preventing them access to mental healthcare services [25, 26]. When access to healthcare or health consequences brought about by treatment (health outcomes) are varying among populations [27], there is a potential for health disparities [28]. Accessing services may be more complicated for adults with intellectual disability due to communication issues and limitations in social support [29]. Individuals who have more severe levels of intellectual disability may find it difficult to communicate their requirements and needs [30]. In addition, challenging behavior has been described as behavior that may drastically restrict or deny the individual from accessing otherwise open community facilities [31]. Challenging behaviors have also been reported by healthcare professionals to be a barrier when providing services to people with intellectual disability [32]. Recognizing mental illnesses in this particular demographic is a multifaceted matter that relies on variables that include the level of intellectual disability, the clinical competence of mental healthcare specialists, the existence or nonexistence of complicating factors like challenging behaviors, and additional factors like the location of residence and the structure of the healthcare services [33].

Frequent use of psychiatric services has been found to be associated with the use of psychotropic medication [34]. The use of psychotropic medication is common among people with intellectual disability, challenging behavior and mental health problems [35, 36] and other interventions from mental health services may be considered less often [37]. Furthermore, a previous Norwegian study found the presence of a challenging behavior to be a common indication of psychotropic prescriptions and that as few as half of all psychotropic prescriptions were indicated by a diagnosis of mental illness. Further, assessments of medications and adverse reactions by psychiatrists were scarce, and therapeutic alternatives were seldom considered [38].

There appears to be a lack of recent studies examining factors associated with the use of mental health services by adults with intellectual disability [24] and previous Nordic studies have mainly focused on older people [39–41]. This may hinder the establishment and assessment of policies and measures to enhance effective service use. The purpose of this study was to address the knowledge gap regarding the use of mental healthcare services among adults with intellectual disability in Norway.

## Methods

### Design, setting and procedure

The North Health in Intellectual Disability (NOHID) study was a cross-sectional multicenter study conducted in Norway. Data collection occurred from October 2017 to December 2019 in the municipalities of Trondheim, Malvik, Narvik, Balsfjord and Tromsø and preceded the outbreak of the COVID-19 pandemic in Norway. The potential participants were identified via the specialized intellectual disability services records at the University Hospital of North Norway (UNN) and St. Olavs hospital, and by acquiring information on adults with intellectual disability who were receiving services from the municipalities [42–44].

All participants and/or their authorized representatives provided informed consent in writing. Data was collected using questionnaires and structured interviews administered to participants and/or their closest family member, carers, or support personnel. The majority of our cases (98%) included proxy responders. In 52% of the cases only the proxy was present. The proxy responders were family or support professionals who had extensive knowledge of the participant. Family members were involved in 62% of the cases, while direct support professionals who had known with the participant for at least one year were involved in 36% of the cases. The medical records of the participants verified information on level of intellectual disability and additional medical conditions [42–44].

The Data Protection Officer at UNN and St. Olavs Hospital and the Regional Committee for Medical Research Ethics (2017/811) granted approval for the study. The study was registered in Clinical Trials with identification number: NCT05703503, registration date was 02/01/2023.

### Participants

Eligible participants were 16 years or older and had a confirmed diagnosis of intellectual disability in accordance with the International Statistical Classification of Diseases and Related Health Problems-10 criteria [45] and resided in the specified municipalities. The living conditions of the participants were classified into two categories: (1) living independently or with family, and (2) living in a group home with care [46].

As presented in Table 1, a sample of 199 participants [55% men, mean age 36.3 (SD 13.9) years] were included in the present study. Participants’ ages ranged from 16 to 78 years. The distribution of levels of intellectual disability among the participants was as follows: mild (39%), moderate (27%), severe/profound (32%), and unknown (2%). Furthermore, 20% of the participants had a diagnosis of Down syndrome, 23% of epilepsy, 22% of autism spectrum disorder, and 13% of cerebral palsy. Of the 199 participants, 22% resided in rural areas, and 78% resided in urban areas. Further, 69% of the participants lived in a group home receiving care services, and 31% lived with family or independently.

**Table 1:** Population characteristics (*N* = 199)

Characteristics	Total, *N* = 199
Age (years)	
Mean (SD)	36.3 (13.9)
Median (range)	33 (16–78)
Gender, *n* (%)	
Women	90 (45)
Men	109 (55)
Level of intellectual disability, *n* (%)	
Mild	78 (39)
Moderate	53 (27)
Severe/ profound	63 (32)
Unknown	5 (2)
Down syndrome, *n* (%)	39 (20)
Autism diagnosis, *n* (%)	43 (22)
Cerebral palsy, *n* (%)	25 (13)
Epilepsy, *n* (%)	45 (23)
Place of residence, *n* (%)	
Rural	44 (22)
Urban	155 (78)
Living condition, *n* (%)	
Group home with care	138 (69)
Lives with family or independently	61 (31)
No organized daily activity, *n* (%)	46 (23)
Reported mental health problems, *n* (%)	47 (24)
Psychotropic medication, *n* (%)	76 (39)
MPAS-Check (above threshold), *n* (%)	36 (18)
ABC-C (overall prevalence), *n* (%)	32 (16)
Respondents, *n* (%)	
Self-report	4 (2)
Self-report and proxy	92 (46)
Proxy	103 (52)
Proxy respondents, *n* (%)	
Family member	121 (62)
Healthcare professional	70 (36)
Other	4 (2)

Note: Psychotropics were defined as any medication that affects behavior, mood, thoughts, or perceptions, not including antiepileptics and analgesics. ABC-C (overall prevalence): participants with one or more items scored as a severe problem on the ABC-C

### Healthcare services, psychotropic medication, and diagnosis

The POMONA-15 (P15) health indicators were internationally developed to assess the extent of health inequalities between people with intellectual disability and the general population [47]. The P15 has been translated to Norwegian and validated [47]. The translated questionnaire used in the present study has previously been published [48]. The P15 were employed to register healthcare service utilization, conditions of autism spectrum disorder, epilepsy, cerebral palsy, Down syndrome, age, gender, place of residence and living condition. Mental health problems were reported during the interview in response to an open question concerning current medical health conditions. Reported sleep disorders, including insomnia and hypersomnia, were registered as mental health problems, while sleep apnea was not included. Data concerning use of medication was gathered using an additional questionnaire. The present study used the Anatomical Therapeutic Chemical (ATC) classification system recommended for drug utilization research by the World Health Organization (WHO). Therefore, medications were categorized based on their interaction with systems or organs, as well as their chemical, pharmacologic, and therapeutic properties. The present study defined psychotropics as any medication that affects mood, perception, thoughts, or behavior, excluding antiepileptics and analgesics. Nevertheless, in accordance with previous research [49], the use of mood-stabilizing antiepileptic drugs was included if the participant did not have epilepsy. The registered psychotropic drugs used were antipsychotics, anxiolytics, antidepressants, mood stabilizers, stimulants, and hypnotics.

### Specialized mental healthcare services

The present study examined the use of mental health professionals, such as psychologists and psychiatrists, as well as specialized habilitation services. Specialized habilitation services for adults in Norway primarily aim to support individuals with complex and lifelong disabilities. These services are typically delivered by multidisciplinary teams, often including physical therapists, psychologists, psychiatrists, disability nurses, and medical doctors, that operate both in outpatient and inpatient settings. A key function of these teams is to provide direct clinical care to patients, as well as indirect support through guidance and consultation to municipal healthcare providers. This dual approach helps ensure continuity of care and enhances the capacity of local services. However, there can be considerable variation in the availability of services, the specific expertise within teams, and the methods used to deliver guidance and support to municipal services.

The questionnaire items presented to the participants were: (1) “Approximately how many times have you been to a psychologist, psychiatrist, or other similar doctor during the last 12 months? (scale/cannot answer)” and (2) “Have you received any follow-up by the specialized habilitation unit during the last 12 months? (yes/no/cannot answer)”. For analysis purposes, item (1) was dichotomized into: “Have you been to a psychologist, psychiatrist, or other similar doctor during the last 12 months? (yes/no)”. To assess the overall use of these services, a new variable was created by combining the use of mental healthcare services and specialized habilitation services. This new binary variable, “Use of mental healthcare and/ or specialized habilitation services in the last 12 months”, was used in the analyses.

### Instruments

The Aberrant Behavior Checklist-Community (ABC-C) was created as a proxy measure to evaluate aberrant/ challenging behavior in individuals with intellectual disability [50]. The ABC-C consists of 58 items that are evaluated on a four-point scale ranging from (0), indicating no problem, to (3), indicating a severe problem. When behavior is reported as a severe problem on the ABC-C, it aligns with the definition of challenging behavior provided by E Emerson, C Kiernan, A Alborz, D Reeves, H Mason, R Swarbrick, L Mason and C Hatton [51]. The binary variable: ABC-C (overall prevalence), see Tables 2 and 3, was created to present the prevalence of challenging behaviors found by the ABC-C. Participants that were reported to have one or more behaviors rated as a severe problem were included in the group presenting challenging behaviors. The items on the checklist are grouped into five subscales: *Irritability*, *Social Withdrawal*, *Stereotypic Behavior*, *Hyperactivity/Non-compliance*, and *Inappropriate Speech*. The Norwegian version of the ABC-C has been found to have satisfactory internal consistency, factor structure, and convergent and divergent validity in earlier studies [52].

**Table 2:** Bivariate associations between demographics, medication, behavior, health conditions, and use of mental healthcare and/ or habilitation services during the last 12 months (*N* = 199)

Characteristics	No use *N* = 101 (51%)	Use *N* = 98 (49%)	*p*
Age (years)			
Mean (SD)	36.3 (13.7)	36.4 (14.1)	0.968
Median (range)	33 (17–78)	33 (16–73)	
Gender, *n* (%)			0.445
Women	43 (48)	47 (52)	
Men	58 (53)	51 (47)	
Level of intellectual disability, *n* (%)			0.239
Mild	37 (47)	41 (53)	
Moderate	32 (60)	21 (40)	
Severe/ profound	29 (46)	34 (54)	
Down syndrome, *n* (%)	25 (64)	14 (36)	0.063
Autism diagnosis, *n* (%)	23 (53)	20 (47)	0.685
Cerebral palsy, *n* (%)	14 (56)	11 (44)	0.575
Epilepsy, *n* (%)	23 (51)	22 (49)	0.957
Place of residence, *n* (%)			0.819
Rural	23 (52)	21 (48)	
Urban	78 (50)	77 (50)	
Living condition, *n* (%)			0.530
Group home with care	68 (49)	70 (51)	
Lives with family or independently	33 (54)	28 (46)	
No organized daily activity, *n* (%)	23 (50)	23 (50)	0.907
Reported mental health problems, *n* (%)	18 (38)	29 (62)	0.051
Psychotropic medication, *n* (%)	34 (45)	42 (55)	0.146
MPAS-Check (above threshold), *n* (%)	11 (31)	25 (69)	0.009
ABC-C (overall prevalence)*, *n* (%)	6 (19)	26 (81)	< 0.001
MPAS-Check, mean (SD)			
Psychotic disorder	0.24 (0.55)	0.54 (1.03)	0.016
Possible organic condition	0.69 (1.40)	1.31 (2.00)	0.016
Affective or neurotic disorder	1.16 (3.12)	2.37 (3.69)	0.020
ABC-C, mean (SD)			
Irritability*	3.21 (4.79)	6.85 (7.51)	< 0.001
Social withdrawal	2.94 (3.78)	3.65 (3.97)	0.198
Stereotypic behavior	0.98 (1.90)	1.57 (2.52)	0.068
Hyperactivity/noncompliance*	2.95 (4.16)	6.58 (7.55)	< 0.001
Inappropriate speech*	0.99 (1.53)	1.97 (2.54)	0.002

Note: Psychotropics were defined as any medication that affects behavior, mood, thoughts, or perceptions, not including antiepileptics and analgesics. ABC-C (overall prevalence): participants with one or more items scored as a severe problem on the ABC-C

* *p*-Values < 0.05 after Hommel correction

**Table 3:** Factors associated with use of mental healthcare and/ or habilitation services the last 12 months in multivariate regression analysis (*N* = 187)

Characteristic	*OR*	*95% CI*	*p*
Age	0.99	0.97–1.01	0.421
Gender:			
Women	0.79	0.42–1.50	0.477
Level of intellectual disability			
Mild			
Moderate	0.92	0.41–2.07	0.832
Severe/ profound	1.07	0.46–2.47	0.882
Down syndrome	0.54	0.23–1.31	0.174
Autism	0.52	0.22–1.22	0.131
Epilepsy	0.91	0.40–2.06	0.818
Psychotropic medication	1.07	0.52–2.19	0.857
MPAS-Check (above threshold)	2.61	1.12–6.07	0.026
ABC-C (overall prevalence)*	4.98	1.80–13.80	0.002

Note: Psychotropics were defined as any medication that affects behavior, mood, thoughts, or perceptions, not including antiepileptics and analgesics

ABC-C (overall prevalence): participants with one or more items scored as a severe problem on the ABC-C

Mild intellectual disability is the reference category for the participants` levels of intellectual disability

* *p*-Values < 0.05 after Hommel correction

The MPAS-Check, referred to as the PAS-ADD Checklist in previous studies, is a proxy measure created for identifying potential mental health issues in individuals with mild to profound levels of intellectual disability. The MPAS-Check scores were obtained through structured interviews, during which the structured questionnaire was administered. The MPAS-Check primarily focuses on identifying current symptoms of mental health issues, emphasizing symptoms present at the time of the assessment. Three subscale scores are computed: *Affective/Neurotic Disorder*, *Possible Organic Condition*, and *Psychotic Disorder*. Each of these subscales has a threshold score specified in the manual. Scores equal to or over this threshold indicate the need for additional mental health examination [53]. The binary variable: MPAS-Check (above threshold), see Tables 2 and 3, was created to present the prevalence of mental health problems indicated by the MPAS-Check. Participants that had scores at or above the threshold, in one or more of the three subcategories, were included in the group with possible mental health problems. The internal consistency of the MPAS-Check was deemed adequate based on the replication of its psychometric properties through independent means. In addition, the MPAS-Check demonstrated sensitivity to variations among diagnostic groups, with an overall sensitivity rate of 66% and a specificity rate of 70% [54].

### Data analysis

Descriptive statistics were employed to provide a comprehensive summary of the data. Continuous variables are reported as means with standard deviations (SD) and median with range was included for the variable of age. Categorical variables are displayed as percentages of the specified categories. The study examined associations with use or no use of services using an independent t-test for the continuous variable of age, Pearson chi-square tests for categorical variables and univariate logistic regression for non-normally distributed scales (MPAS-Check subscales and ABC-C subscales).

In addition, a multivariate logistic regression analysis was conducted to improve the interpretation of data. By default, multivariate regression analysis excludes cases that have missing data on any variable. Consequently, 12 participants were excluded from the final logistic regression analysis (*n* = 187). A dummy variable was created from the three levels of intellectual disability on an ordinal scale with “mild intellectual disability” as the reference category. The statistical analysis was conducted using IBM SPSS Statistics version 29.0.1.0.

Multiple verifications were carried out to confirm that the necessary conditions for logistic regression analysis were fulfilled. The number of events per independent variable was sufficient, and there were no strongly influential outliers that may alter the results and accuracy of the model. Cross-tabulations were used to examine nominal variables, ensuring that the expected counts were sufficient. The linearity assumption of independent continuous variables and associated logit/log-odds was verified. The variance inflation factor (VIF) values for all included independent variables were less than 3, indicating a low level of multicollinearity in the regression model. The enter method was used.

To assess the relationship between the independent variables: age (scale), gender (male/female), level of intellectual disability (ordinal scale 1–3), Down syndrome (yes/no), autism (yes/no), epilepsy (yes/no), psychotropic medication use (yes/no), MPAS-Check (above threshold), ABC-C (overall prevalence), and our dependent binary variable of service use, a multivariate logistic regression analysis was conducted. The effect sizes of the independent variables are reported as odds ratios (OR) with 95% confidence intervals. The level of significance was set at α = 0.05. The present study incorporates multiple analyses, making it susceptible to false-positive results. The Hommel correction was applied to all findings to control family-wise error rate. The final model fit was assessed with the Hosmer and Lemeshow test, and the degree of pseudo-explained variance was reported according to Nagelkerke R^2^.

## Results

### Participant characteristics

Reported mental health problems were identified in 24% of the 199 participants. The mental health problems identified in the sample were depression, anxiety, bipolar disorder, schizophrenia, organic mental disorder, unspecified affective disorder, post-traumatic stress disorder, attention deficit hyperactivity disorder, and sleep disorders. Psychotropic medications were used by 39% of the participants. Characteristics of the participants are presented in Table 1.

The present study did not find a significant association between use of psychotropic medication and the utilization of mental healthcare and/or specialized habilitation services the last 12 months. Worth noting is that 45% of the participants currently using psychotropics did not see a mental health care specialist or use the specialized habilitation services the last year.

### ABC-C and MPAS-check

As indicated by the MPAS-Check, the overall prevalence of mental health disorders in the study population (*n* = 199) was 18%. These participants had scores at or above the threshold, defined by the MPAS-Check, in one or more of the three subcategories. Of the participants, 13% crossed the threshold for *affective/neurotic disorder*, 5% for *possible organic condition* and 11% for *psychotic disorder*. The highest average score was found on the subcategory indicating the presence of an *affective/neurotic disorder* and the lowest for *psychotic disorder*.

Of the 199 participants, 32 had one or more items reported as a severe problem on the ABC-C. Therefore, the occurrence of challenging behavior in our sample was estimated to be 16%. The constituent items of the subcategory of *irritability* were most frequently rated as severe problems. The subscale representing *stereotypic behavior* had the lowest average score, whereas *irritability* had the highest average score.

### Use of services

Of the 199 participants, 16% had used mental healthcare services (seen a psychologist or psychiatrist or other mental health professional), 48% had used specialized habilitation services and 49% had used mental healthcare or specialized habilitation services during the last 12 months.

Table 2 presents the bivariate associations between participant characteristics, psychotropic medication use, outcomes of the instruments used to assess mental health problems (MPAS-Check) and challenging behavior (ABC-C), and mental healthcare and/or specialized habilitation service use the last 12 months (yes/no) in the study population (*n* = 199). ABC-C (overall prevalence), *X*^*2*^ (1, *n* = 32) = 15.9, *p* < 0.001, *irritability* (unadjusted OR = 1.10, 95% confidence interval (CI) 1.05, 1.17), *hyperactivity/noncompliance* (unadjusted OR = 1.11, 95% CI 1.05, 1.18) and *inappropriate speech* (unadjusted OR = 1.27, 95% CI 1.09, 1.48) were significantly associated with service use after the Hommel correction was applied. Participants that had one or more items rated as a severe problem on the ABC-C, indicating challenging behavior (ABC-C (overall prevalence)), were more likely to use the defined services the last 12 months. Further, the scores on the *Irritability* subcategory of the ABC-C were higher in the group using the services than in the group not using the services. In addition, the scores on the *Hyperactivity/noncompliance* and the *Inappropriate speech* subcategories of the ABC-C were higher in the group using mental healthcare and/or specialized habilitation services the last 12 months.

### Multivariate analysis

Factors associated with the use of mental healthcare and/or specialized habilitation services the last 12 months (yes/no) in multivariate logistic regression analysis are presented in Table 3. The presence of challenging behavior measured by the variable: ABC-C (overall prevalence) (OR = 4.98, 95% CI 1.80, 13.80) was the only significant explanatory variable for use of the specified services after the Hommel correction was applied. Worth noting is that scoring above threshold on the MPAS-Check, indicating the presence of mental health problems (OR = 2.61, 95% CI 1.12, 6.07), significantly increased the odds of using the services. However, the variable did not stay significant after controlling for family-wise error rate with the Hommel correction. The Hosmer and Lemeshow test indicated a good model fit (*χ*^2^ 12.70, *df* = 8, and *p* = 0.123). The Nagelkerke *R*^2^ was 0.168.

## Discussion

### Main study findings

The present study described the use of mental healthcare and specialized habilitation services during the last 12 months among a sample of 199 community-based adults with intellectual disability in Norway. Furthermore, the contribution of this study was to address the knowledge gap regarding how the use of these services is associated with mental health problems, challenging behavior, and other relevant factors. About half (49%) of the 199 participants had used mental healthcare and/or specialized habilitation services during the last 12 months. Specifically, 16% had used mental healthcare services (seen a psychologist or psychiatrist) and 48% had used specialized habilitation services. Of the 199 participants, 24% reported having mental health problems and the overall occurrence of mental health problems found by the MPAS-Check was 18%. The occurrence of challenging behavior in our sample was found to be 16% using the ABC-C. The presence of challenging behavior was found to significantly increase the odds of using mental healthcare and/or specialized habilitation services.

### Use of services

The present study found that, during the last 12 months, 16% of the participants had received services from a psychologist, psychiatrist, or other mental health professional. To our knowledge, no recent studies providing detailed comparative data on mental health service utilization by adults with intellectual disabilities in Norway are available. However, a comparable study from Canada found that 18% had received services from a psychiatrist and 7% from a psychologist in the last year [19]. A Swedish study found that people with intellectual disability were three times as likely to use psychiatric services, compared to an age- and gender-matched sample from the general population over a 10 year period [39]. Similarly, a Swedish national register study revealed that psychiatric health care use was higher among people with intellectual disability than in the general population after adjusting for age, gender and co-morbidities [40]. Still, previous international studies indicate that the increased prevalence of mental health problems among people with intellectual disability [5, 25] does not correspond with their use of mental health services, which remains insufficient [24, 26, 55]. In contrast, the present study found that 49% of participants used mental healthcare and/or specialized habilitation services, while 24% reported mental health problems. A possible limitation is that the specialized habilitation services provide physical as well as mental health care to people with intellectual disability. This may complicate comparing the results of the present study with international findings.

The overlap between general mental health services and specialized habilitation services is evident in the present study. Of the 32 participants treated by general mental health services in the previous year, 91% (29 participants) also used specialized habilitation services. Thus, 48% of the participants had used specialized habilitation services and 49% had used mental healthcare and/or specialized habilitation services during the last 12 months. The higher utilization of specialized habilitation services among the participants may reflect the broader approach of these services. Additionally, adults with moderate, severe, and profound intellectual disabilities are rarely treated by general mental health services in Norway. It is likely that most of these patients are referred to specialized habilitation services, but primarily due to issues other than mental health, such as challenging behaviors [56]. In 2021, more than 10,000 adult patients received habilitation services in the specialist health service in Norway [57].

In the present study, challenging behavior was the only significant explanatory variable. Participants with challenging behavior had close to five times higher odds of using the services compared to participants without challenging behavior. Although the presence of mental health problems, as indicated by the MPAS-Check screening instrument, also initially showed a significant association with service use, this did not remain significant after adjusting for family-wise error rate.

These results are consistent with previous studies from the Nordic countries where challenging behavior was found to increase the frequency of both inpatient and unscheduled visits to psychiatric healthcare [41] and mental health problems and behavioral disorders were associated with increased use of psychiatric health care [40]. These findings may be consistent with expectations since challenging behavior has previously been seen as a behavioral manifestation of psychiatric disorders [58]. Conversely, research suggests that challenging behavior and psychiatric disorders may be distinct and separate conditions [59, 60]. Various difficult circumstances in life or in the relationship to others, such as insecure attachment, can lead to challenging behavior [61].

Previous studies have identified factors associated with the use of mental health services by people with intellectual disability on an organizational as well as personal level [24]. Most people with intellectual disability are under close supervision by support staff who are likely to help facilitate their access to mental healthcare services when necessary. In the present study, 69% of the 199 participants were living in a group home with care and 77% of the participants had an organized day activity in the form of day care or sheltered employment. However, living conditions and having an organized day activity were not associated with the use of mental healthcare and/or specialized habilitation services. While existing research is limited and presents conflicting results, certain studies offer valuable insights into this area. In the UK, people with intellectual disabilities who utilized psychiatric services tended to be older and were more frequently living in residential settings [62]. Conversely, a previous Nordic study found that people living in housing with special services were less likely to use mental health services [39].

Diagnostic overshadowing is a well-documented phenomenon where symptoms of physical or mental health conditions are misattributed to a person’s intellectual disability, leading to underdiagnosis or misdiagnosis of treatable conditions [63]. This issue is exacerbated by the significant overlap in symptoms between mental health issues and intellectual disabilities, making it challenging to distinguish between them [64]. There is a potential for diagnostic overshadowing, as described by S Reiss [65], if support and health care staff attribute challenging behavior or other symptoms among people with intellectual disability to their disability. Previous studies have pointed out that there may be limited knowledge about challenging behavior and intellectual disability among health care staff [41]. This may reduce the effectiveness of primary healthcare [1], hinder effective identification of mental health problems and thus increase the use of mental healthcare and/or specialized habilitation services. Furthermore, it is crucial to note that alterations in behavior could potentially be indicative of an undiagnosed somatic disease or other illness [66]. Further enhancing the competence of staff providing daily services to people with intellectual disability and mental health problems may facilitate improved efficiency and access to mental health services [23, 67].

In the present study, 39% of the respondents reported the use of psychotropic medication, which is higher than the 24% who reported mental health problems and the 18% identified by the MPAS-Check screening instrument. These findings aligns with the results of a UK population-based study from 2015 [36]. This may suggest that factors other than mental health problems influence psychotropic medication use, or there may be a possible underrecognition of psychiatric disorders [68].

The use of psychotropic medication was not associated with the use of mental healthcare and/or specialized habilitation services in the present study. Worth noting is that 45% of the 76 participants currently using psychotropics did not see a mental health care specialist or use the specialized habilitation services in the last year. A previous Norwegian study reported that psychotropic medication was commonly prescribed to people with intellectual disability without the consultation of a psychiatrist. The same study found that prescriptions often deviated from existing guidelines, particularly when issued by general practitioners [38]. Although being far beyond what can be concluded in the present study, a potential use of psychotropics without regular medication reviews is problematic and may result in substantial harm to the patient [69, 70]. Furthermore, studies have found that prescriptions of psychotropics are frequently indicated by challenging behavior [38, 71]. Research points to the need for multidisciplinary teams, including general practitioners, psychiatrists, and other healthcare professionals, to identify the underlying mechanisms of mental health problems or challenging behavior and to suggest psychosocial and behavioral treatment as potential alternatives to the prescriptions of psychotropic drugs [72]. Collaborating in multidisciplinary teams may contribute to knowledge development for all involved healthcare professionals.

### Limitations

The NOHID-study had a cross-sectional design, making it challenging to determine a causal direction of the association between healthcare utilization and factors like mental health, challenging behavior and psychotropics. These factors have the potential to be a consequence as well as a cause of healthcare utilization. Employing a longitudinal design would legitimate further examination of the relationship between these factors and the use of healthcare services in people with intellectual disability.

The demographic representation of our sample may have been influenced by the fact that eligible participants were identified by receiving health services or care. In addition, the analysis of representativity found that, compared with eligible non-participants, the included participants were significantly younger [42–44]. Therefore, the rates of healthcare utilization, mental health problems, and challenging behaviors found in the present study may have been influenced by selection bias and may not be generalizable to all individuals with intellectual disability. General healthcare service utilization by people with intellectual disability in Norway has been found to increase with age [43]. However, the age difference between the group that reported use of mental healthcare and/or specialized habilitation services the last 12 months and the group that did not was minimal in the present study. Furthermore, the rates of mental health problems and challenging behavior found in the present study were coherent with previous findings by JL Taylor, C Hatton, L Dixon and C Douglas [73] and E Myrbakk and S Von Tetzchner [74].

One limitation of the present study is that 98% of the cases involved proxy respondents. The NOHID-study aimed to include all eligible participants, irrespective of communication abilities or cognitive functioning, by prioritizing flexibility in data collection. All participants were given the option to bring a familiar support person with them that could assist with difficult questions and, if preferred, respond on their behalf. While the study aimed to maximize the participation of individuals with intellectual disabilities, it also allowed for partial or full representation by a close caregiver or family member during data collection. Due to considerable individual differences, people with mild intellectual disability may answer questions independently, while those with profound intellectual disability more often need a support person to respond on their behalf [75]. Still, self-report is generally preferred over proxy reports [76, 77]. This preference stems from ethical considerations [78] and methodological concerns, as proxy reports can be less sensitive and accurate [77, 79, 80]. This affects the validity of responses in the present study, especially for mental health-related questions, since proxies may not fully grasp the internal mental state of the participants. This approach may not capture the full scope of mental health issues as accurately as direct clinical evaluations or self-report measures. Nevertheless, many variables in the present study concentrated on objective data, including gender, age, and the use of healthcare services, which are less susceptible to observer bias. Additionally, information on levels of intellectual disability and comorbid conditions, such as epilepsy and Down syndrome, was verified by accessing participants’ medical records [42].

A new variable was created to assess the overall use of mental health or specialized habilitation services in our sample. The new variable was created by collapsing the variables measuring the use of mental health care services and specialized habilitation services. A possible limitation is that the specialized habilitation services provide physical as well as mental health care to people with intellectual disability. This may complicate comparing the results of the present study with international findings.

The living conditions of the participants were classified into two categories: (1) living independently or with family, and (2) living in a group home with care. A limitation of the present study is the merging of “living independently” and “living with family” into a single category. These two living arrangements represent very different circumstances that can have distinct impacts on mental health and use of services. By merging these categories, important nuances in how these living arrangements affect the use of services may have been overlooked. Future research should consider analyzing these categories separately to better understand their distinct impacts. However, the group of adults with intellectual disabilities living independently was relatively small (*n* = 19) in the present study, and merging these categories allowed for maintaining a sufficient sample size for statistical analyses and ensuring the robustness of the findings. Additionally, living in a group home with care represents a distinct environment characterized by structured support and professional care. This setting often includes regular supervision and assistance with daily activities, which can significantly differ from the experiences of those living independently or with family.

Various predictors, such as socioeconomic status, may be associated with use of health care services and were not adjusted for in the study. However, Norway has universal health coverage financed by taxation, and the welfare policy upholds that all citizens are entitled to basic healthcare services [14].

## Conclusion

About half (49%) of the 199 participants had used mental healthcare and/ or specialized habilitation services during the last 12 months. Specifically, 16% had used mental healthcare services (seen a psychologist or psychiatrist) and 48% had used specialized habilitation services. The presence of challenging behavior was found to significantly increase the odds of using mental healthcare and/or specialized habilitation services. Despite frequent use of psychotropic medication among the participants, the results of the present study may indicate that the use of these medications is not followed up by mental healthcare or specialized habilitation services regularly. This underscores the need for regular medication reviews and adherence to guidelines by multidisciplinary teams to ensure safe and effective prescribing practices.

## Data Availability

No datasets were generated or analysed during the current study.
